# Application of Metal Shielding Materials to Protect Buildings Occupants from Exposure to the Electromagnetic Fields

**DOI:** 10.3390/ma16155438

**Published:** 2023-08-03

**Authors:** Ervin Lumnitzer, Elena Lukac Jurgovska, Miriam Andrejiova, Ruzena Kralikova

**Affiliations:** 1 Department of Business Management and Environmental Engineering, Faculty of Mechanical Engineering, Technical University of Košice, 040 01 Kosice, Slovakia; ervin.lumnitzer@tuke.sk; 2 Department of Applied Mathematics and Informatics, Faculty of Mechanical Engineering, Technical University of Košice, 040 01 Kosice, Slovakia

**Keywords:** electromagnetic field, shading, metal material, electric field strength, ANOVA

## Abstract

In recent decades, the background level of electromagnetic fields (EMFs) has increased extremely. One of the decisive factors influencing this increase is the increase in the quality, volume, and speed of voice and data services of mobile operators. This paper deals with the protection of the internal environment from the negative effects of EMFs through elements made of metal materials that absorb this radiation. For the purposes of this research, a series of measurements were carried out on individual days of the week and hours during the day. The results of the measurements were evaluated by the ANOVA method. The aim was to obtain a summary overview of the effects of electromagnetic fields and propose measures for their elimination in the interior. Therefore, measurements of electromagnetic fields were also carried out using shielding elements made of various metal materials, and a comparison of their shielding efficiency was subsequently made. Applications of shading blinds with the highest shading efficiency were recommended to increase safety, protect people’s health from its effects, and prevent electromagnetic fields.

## 1. Introduction

Since the beginning of the 20th century, we have been overwhelmed by the increasing sources of electromagnetic fields (EMFs) coming from telecommunication, electricity, appliances, medical equipment, and many other apparatuses that we use in our daily life [1]. As societies develop, greater use of certain technologies leads to increasing exposure to static electric and magnetic fields. Electromagnetic fields of all frequencies represent one of the most common and fastest growing environmental influences, about which there is anxiety and speculation is spreading. These are also current in the residential environment, so a new term is encountered in the specialty literature “electrosmog”. It includes all artificial electromagnetic emissions (created by humans) and is becoming more and more of a major public health concern [2]. Some materials prevent the transmission of electromagnetic (EM) radiation by reflection and/or absorption of the electromagnetic radiation or by suppressing the EM signals. In general, conductive materials like metals, owing to their high reflectivity, are widely used to isolate spaces or equipment from surrounding EM waves. This reflection shielding is based on the principle of the Faraday cage, in which inside the cage, space is completely impervious to external electric fields. Electrostatic shielding is the phenomenon that is observed when a Faraday cage operates to block the effects of an electric field. Such a cage can block the effects of an external field on its internal contents, or the effects of an internal field on the outside environment [3]. The potential effects of electromagnetic fields on human health vary widely depending on the frequency and intensity of the fields [4]. The space around an electric charge in which its influence can be felt is known as the electric field [5].

The electric field intensity at a point is the force experienced by a unit of positive charge placed at that point. The electric field intensity (E) is a vector quantity; it has magnitude as well as direction and has units of electrical potential per distance (V/m). Human exposure to electromagnetic fields (EMFs) is on the rise and various health problems are increasing. Electromagnetic shielding strategies are increasingly incorporated in the construction of new buildings and are easily retrofitted in existing constructions to reduce radiation intensity in living or working spaces. There are various types of shielding used in the reduction in radiation exposure including lead glasses, lead barriers, conductive shading paints, window films, shade nets, and blinds [6]. Exposure of people to the totally polarized EMFs of human technology, especially Radio Frequency (RF) or microwave (in the GHz range) and Extremely Low Frequency (ELF) (0–3000 Hz) EMFs from Mobile Telephony (MT) antennas, and ELF 50–60 Hz electric and magnetic fields from power lines, has increased to unprecedented levels in order to satisfy the increasing demands of technological applications used by the modern society [7,8,9]. Indeed, any metal shielding practice, even when correctly applied, attenuates not only man-made totally polarized EMFs accused of health problems but also the natural non-polarized EMFs responsible for the biological rhythmicity and well-being of all animals. Strong evidence of this was provided by pioneering experiments in the 1960s and 1970s, with volunteers staying in a shielded underground apartment [10,11,12,13]. Effects of man-made electromagnetic fields (EMFs) on living organisms potentially include transient and permanent changes in cell behavior, physiology, and morphology [14]. In [15], the authors discussed three different measurement cases from fifth generation base stations. Total E-field strength in all cases is no more than 91 dB mu V/m. Pophof et al. [16] investigated the biological effects of RF-EMF on fauna and flora. This area is not well studied. For high frequencies exceeding 100 MHz, the only scientifically established action mechanism in organisms is the conversion of electromagnetic into thermal energy. In accordance with that, no proven scientific evidence of adverse effects in animals or plants under realistic environmental conditions has yet been identified from exposure to low-level anthropogenic radiofrequency fields in this frequency range. In an article by Tomitsch et al. [17], measurements of exposure to electric, magnetic or electromagnetic fields (EMFs) in households were performed. The article reported that all measurement results were well below the International Commission on Non-Ionizing Radiation Protection (ICNIRP) guidelines. Similarly, in [18], authors conducted an experiment comparing the radiated power levels of base stations with the ICNIRP safety guidelines. The measured EMF levels were lower than the ICNIRP exposure limits. In the publication [19], the authors proposed a simple electric and magnetic field shielding evaluation method of board-level shields. The measured shielding effectiveness was validated by numerical simulation and thus reliable results were obtained.

Therefore, people are advised to protect themselves from man-made EMFs by metal shielding through various products, for which there are reasonable concerns about their protective efficacy and safety. Since metal shielding has lately been massively suggested and applied as a protective solution against man-made EMFs, it is necessary to take scientific verification by the in-practice measurements. Methods to measure the electromagnetic field intensity on a large scale should be improved to meet the growing demand in civil and industrial applications [20].

The present article relates the methodology and methods for the measurement of electromagnetic fields generated by mobile operator base stations. In the case of assessing the impact of base stations on humans, it is an assessment of high-frequency electromagnetic fields. The basic parameter established by the Slovak legislation [21] and therefore used in this evaluation is the electric field strength.

## 2. Materials and Methods

The issue of contribution is aimed at absorbing the external high-frequency electromagnetic fields with metallic materials. The absorption principle is based on the Faraday cage, which consists of concentrating the electrical charge only on the surface and not on the volume of the structure. The design of blinds used is designed to create a mutual rocking motion between the blinds in the direction of the EMF propagation. For this reason, there is minimal penetration of EMFs through the blinds. The synergy also lies in the combination of the Faraday cage as the driver and the absorption of the electric charge by the top of the structure.

The contribution can be divided into two parts. In the first part, a statistical evaluation of the measured data is carried out during the whole week in a two-hour interval. The evaluation determined the day and hour when the highest intensity of the electric field was measured in the selected location with the selected source. For this research, we chose a base transceiver station as the main source of the electromagnetic field in the evaluated space. For the purposes of the presented research, the measurement and evaluation methodology are used to assess changes in EF strength in variety according to the time of the day (from 8 a.m. to 8 p.m.), the day of the week (measurements on each day of the week) and on the measurement site in the selected location.

In the second part of the paper, the exact type of shape and material solution for shields that is available on the market is presented. We see its use as justified also in solving the problem of shielding buildings from the effects of electromagnetic fields on residents.

**Figure 1:**
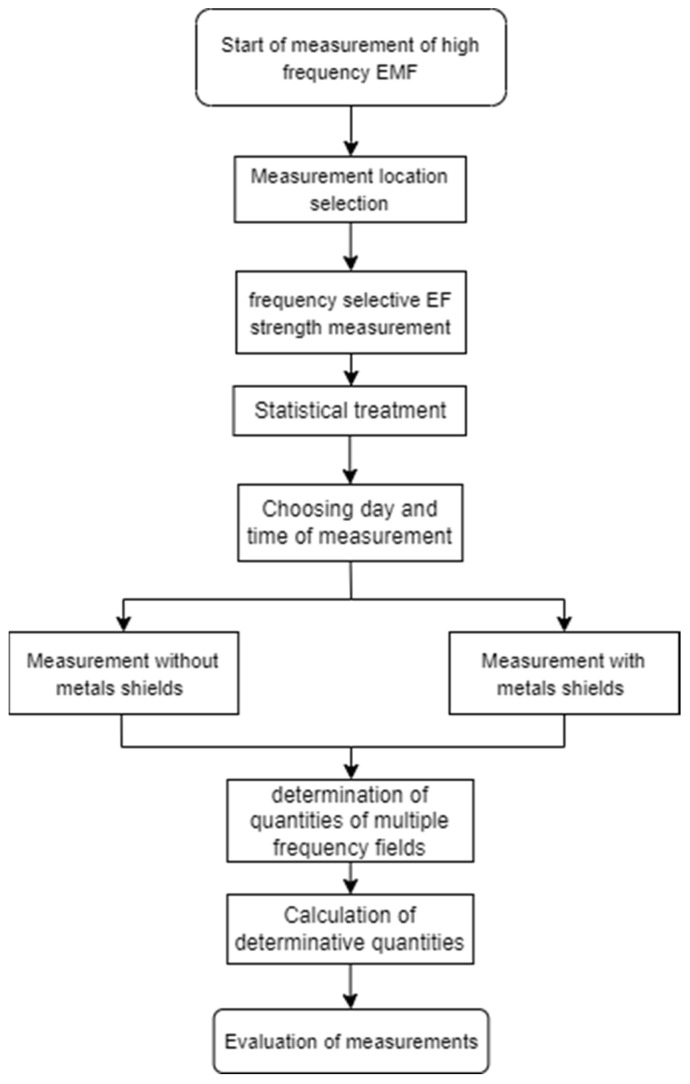
Diagram of measurement methods.

The main purpose of the document is to identify changes in the intensity of the electric field in the interior of the building using metal shields. A simplified diagram of the methodology for handling the contribution is shown in Figure 1.

### 2.1. Mobile Network Base Station

The base station is one of the essential elements forming the terrestrial network for the operation of mobile communications and is currently the strongest source of EMFs in the vicinity of our homes. Base stations transmit and receive low-powered radio signals to and from mobile phones and provide connection to the main telephone network. They need to be located close to mobile phone users to provide good reception [22]. 

Experimental measurements of electromagnetic fields generated by base stations of mobile operators were performed at a selected site. The area is built up of industrial buildings, production halls as well as administrative buildings. Companies in the industrial zone use internet networks and communication systems that are a source of electromagnetic fields. A tram line runs through the area. There is an increased level of movement and work activities at the site during working days. The demand and performance of base stations are lower during weekends. 

The basic parameters of the mobile phone base stations include their location, frequency band used, tilt, and direction of antenna radiation [22,23]. There are several in the chosen location, but for this experiment, the one that is close to the measuring points and its operation can affect the measured values is important.

**Figure 2:**
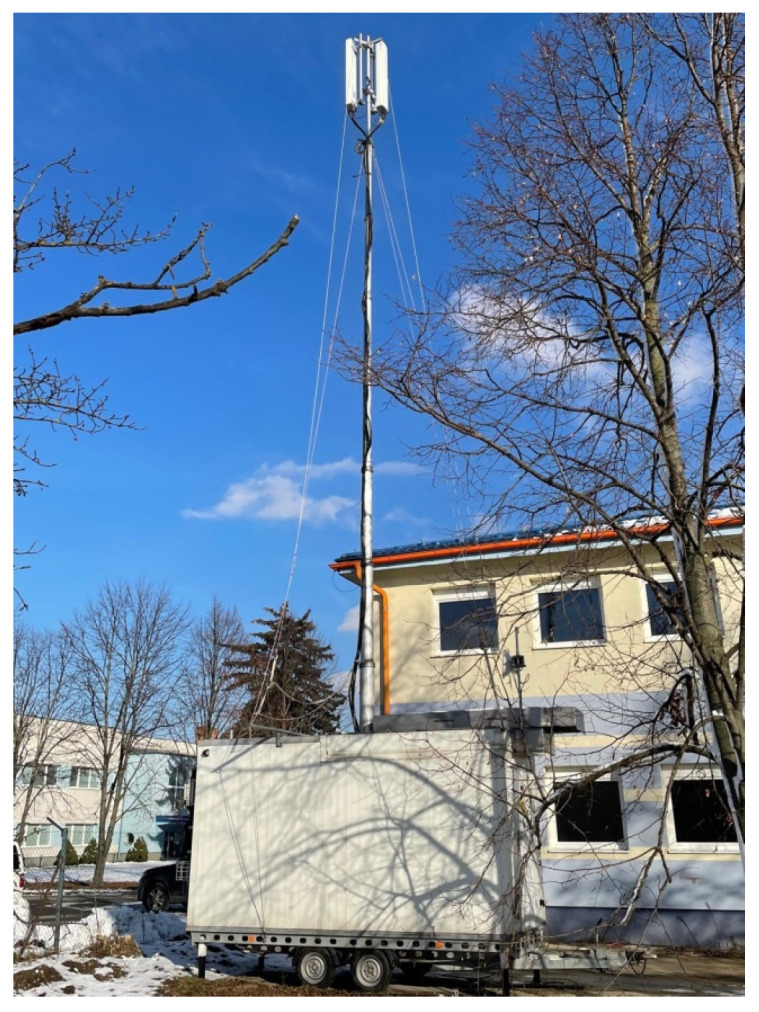
Location of mobile base transmitting station.

The mobile base transmitting station (MBTS) is located next to an occupied building at 68 Juzna trieda Street. This station is operated as a dual sector at a height of 15 m and azimuths of 175° and 345°. MBTS uses all frequency bands, i.e., 800 MHz, 900 MHz, 1800 MHz, 2100 MHz, and 2600 MHz. Figure 2 shows the mobile base transmitting station.

### 2.2. Statistical Methods of Evaluation

Basic statistical methods, descriptive statistics, statistical hypothesis testing, and the ANOVA method were used in the evaluation of the data. In statistical hypothesis testing, the decision to reject or accept the null hypothesis is made using the *p*-value. If the *p*-value is less than the specified significance level α, the null hypothesis is rejected in favor of the alternative hypothesis. If the *p*-value is equal to or greater than the chosen significance level α, we do not reject the null hypothesis. 

#### 2.2.1. Analysis of Variance

Analysis of variance (ANOVA) is a method that allows for comparison of the mean values of several independent baseline sets. The basic assumptions of using analysis of variance include independence (individual samples are independent of each other), normality (samples come from basic files with normal distribution), and homoscedasticity (homogeneity, similarity of variances of basic files).

A Shapiro–Wilk normality test is recommended to verify normality. We test the null hypothesis: “the sample distribution is normally distributed” against the alternative hypothesis “the sample distribution is not normally distributed”.

A suitable test for examining homogeneity of variances is the Bartlett test. We test the null hypothesis “the variances of all sets are equal” against the alternative hypothesis “equality does not apply to at least one pair of variances”.

In our case, we will examine the effect of two factors A and B on the variability of the variable of interest. Factor A has level I and factor B has level J, where the factors do not affect each other and we will consider a two-way ANOVA without interactions. 

For the effect of factor A, we test the null hypothesis $H_{0}^{\alpha }:\alpha_{1}=\alpha_{2}=\cdots =\alpha_{I}=0$ against the alternative hypothesis “at least one of the equations is not met”. By analogy, for factor B, the null hypothesis is $H_{0}^{\beta }:\beta_{1}=\beta_{2}=\cdots =\beta_{J}=0.$ This means that if we accept the null hypothesis at the specified significance level, then factor A (or B) has no statistically significant effect on the variability of the measured value.

As a measure of the magnitude (strength, intensity) of the effect of factor A (or B), we use the $\eta^{2}$ coefficient, which is analogous to the coefficient of determination in regression analysis. For the effect of factor $\eta^{2}$, the relation could be expressed as

(1) $$\eta^{2}=\frac{{SS}_{factor}}{SST},$$

where ${SS}_{factor}$ is the variability between sets explained by the respective factor (for factor A, it is ${SS}_{A}$, and for factor B, it is the variability between the levels of factor B, i.e., ${SS}_{B}$) and SST is the total variability. The coefficient $\eta^{2}$ determines how much data variability is explained by a given factor or an interaction of factors. The coefficient *η*^2^ takes on values from 0 to 1. The closer the value of the coefficient is to 1, the greater the effect of the factor of interest. The value of coefficient $\eta^{2}$ can be interpreted as follows: value $\eta^{2}$ from interval (0.01, 0.059) indicates a small effect, value $\eta^{2}$ from interval (0.06, 0.14) indicates a medium effect, value $\eta^{2}$ greater than 0.14 indicates a large effect. The value of the coefficient is often given as a percentage [24,25].

#### 2.2.2. Multiple Comparisons Tests (Post Hoc Tests)

If we find a statistically significant effect of at least one factor when examining the effect of factors on the observed variable, then using post hoc tests (e.g., Scheffe method) we determine which pairs of levels of that factor differ significantly from each other. We test the null hypothesis “differences in mean values between the pair of sets are insignificant” against the alternative hypothesis “differences in mean values between the observed pair of sets are statistically significant”.

## 3. Results

Experimental research was conducted to:

- map the location and select suitable measurement sites,
- analyze the measured cumulative EF strength values in terms of the measurement site, day of the week and time of the day,
- compare values and identify the dependencies of the EF strength in terms of selected factors (day, time of measurement) by means of appropriate statistical methods,
- analyze the situation of the intensity of the electromagnetic field in the select building,
- propose technical measures against non-ionizing sources.

### 3.1. Selection of Measurement Sites

**Figure 3:**
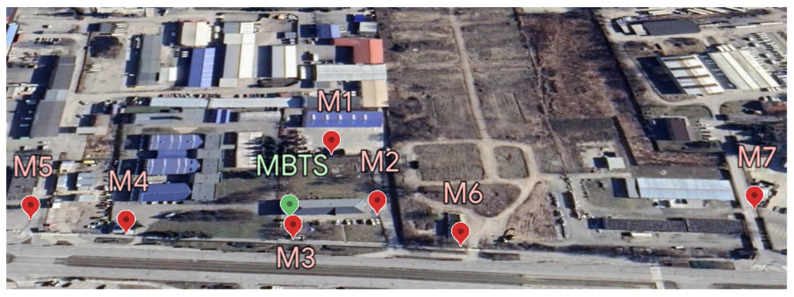
Location of measurement sites.

For the measurements, seven measurement sites, labelled M1–M7, were selected according to the measurement methodology shown in Figure 3. Considering the research objectives, all measurement sites were located in a far-field area. The measurement sites were chosen to characterize the selected location, taking into account the directional characteristics of the antennas. Measurements at all measurement sites were performed at the time of peak load of the base stations from 8 a.m. to 8 p.m. at 2 h intervals throughout the week. 

The distance of each measurement point from the MBTS is shown in Table 1.

**Figure 4:**
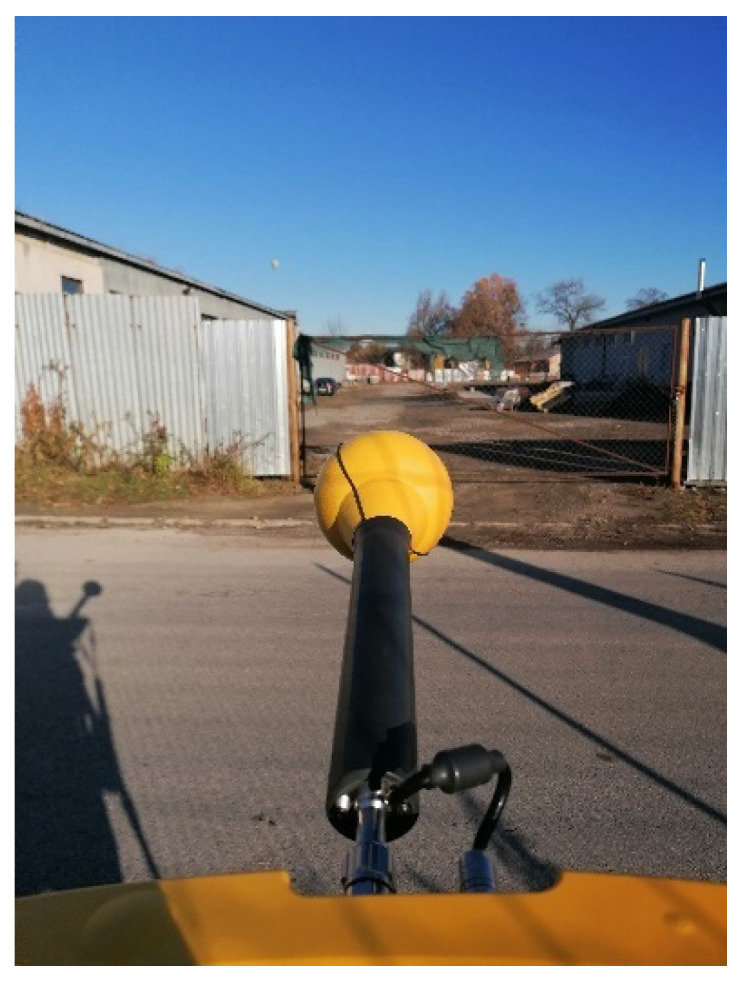
Location of measurement site M7 during measurement.

Measurements were performed at each measurement site using a Narda SRM-3006 measuring instrument consisting of a spectrum analyzer and an isotropic probe for measuring the electric field component. The measuring instrument was placed on a carbon tripod at a height of 1.5 m. The output of the measurements is the frequency spectrum, cumulative values of the electromagnetic spectrum intensity, and the maximum EMF values for the dominant frequencies at each measurement site during individual measurements. The measurement time was set to six minutes. The measurement was performed in the frequency band of the measurement chain from 420 MHz to 6 GHz. The minimum display frequency was F_min_—700 MHz, and the maximum display frequency was F_max_—3500 MHz. Figure 4 shows the location of the measurement site M7 during the measurement. 

### 3.2. Analysis of Measured Values

The main outputs of individual measurements are the cumulative values of electric field strength and frequency spectrum graphs for each measurement. Table 2 shows the cumulative values of EF strength (mV/m) for two selected sites M1 and M7 for the whole measurement period, every two hours from 8 a.m. to 8 p.m. during the whole week. Providing data from all measurement sites would go beyond the scope of the contribution.

The resulting numerical characteristics of the measurements carried out on different days of the week (regardless of the site and time of the measurement) for a given station are shown in Table 3.

**Figure 5:**
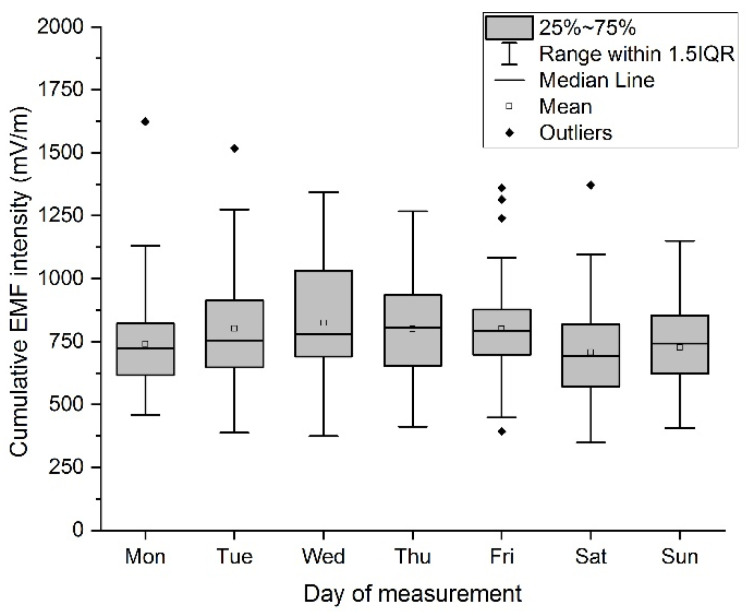
Boxplot of cumulative EF strength value—day of measurement.

The measured values show that the lowest average value was recorded on Saturday. A graphical representation of the values by means of boxplots is shown in Figure 5.

The impact of the day of measurement on the resulting value is characterized by the variance of the measured values depending on the day of the week. From the results, the choice of the day of measurement does not significantly affect the measured values, the most significant decreases were recorded during the weekend on Saturday and Sunday. However, these decreases were not statistically significant.

The resulting numerical characteristics of the measurements performed at different times during the day (regardless of the site and day of the measurement) for a given station are shown in Table 4. 

**Figure 6:**
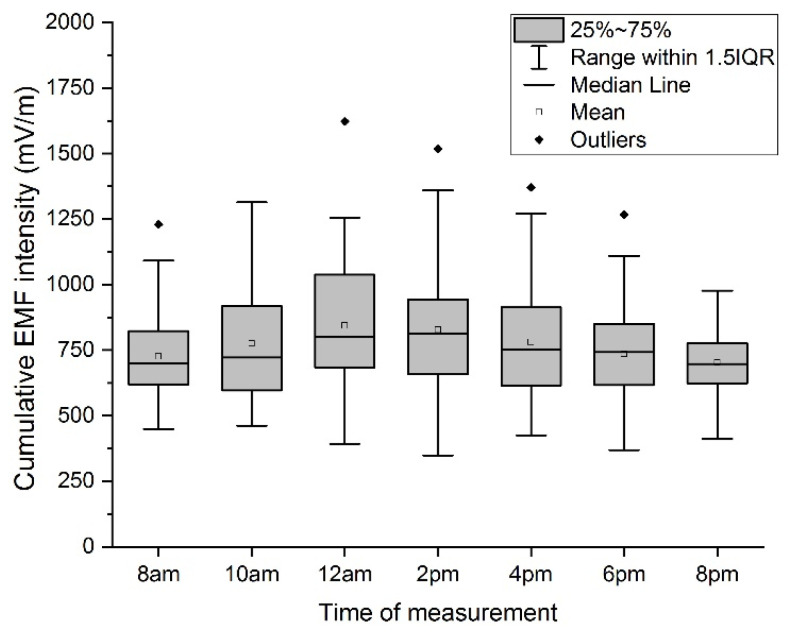
Boxplot of cumulative EF strength value—time of measurement.

The measured values show that the lowest average value was recorded on Saturday. A graphical representation of the values by means of boxplots is shown in Figure 6.

The impact of the time of measurement during the day on the resulting value is characterized by the variance of values measured during individual days. From the results, the choice of time of measurement does not significantly affect the measured values. Generally, the lowest values were recorded in the morning at 8 a.m. and the evening at 8 p.m.. There were occasional local extremes, not corresponding to this statement, which were probably caused by increased data flow at the time of measurement (e.g., running backup data).

### 3.3. Comparison of Values Using the ANOVA Method

We will use hypothesis testing methods and two-way analysis of variance without interactions to compare the measured values of cumulative EF strength. Three factors are considered in the analysis of the measured values: measurement site (seven levels), day of measurement (seven levels), and time of measurement (seven levels), which can significantly affect the measured values (see Table 5). 

In the next section, we will observe the influence of selected factors on the cumulative values of EF strength in three different cases:

- cumulative value of EF strength at the i-th measurement site Mi, factors: day of measurement (factor A) and time of measurement (factor B), where i = 1, 2, …, 7
- cumulative value of EF strength on the i-th day of measurement Di, factors: time of measurement (factor A) and measurement site (factor B), where i = 1, 2, …, 7
- cumulative value of EF strength in the i-th time of measurement Hi, factors: day of measurement (factor A) and measurement site (factor B), where i = 1, 2, …, 7.

In each case, we test the effect of two factors (factor A, and factor B) on cumulative values of EF strength. If we test the effect of factor A (or B), the null hypothesis H0 is: “Factor A (or B) does not affect the final value” against H1: “Factor A (or B) affects the final value”. If the *p*-value is less than the significance level α, then the null hypothesis is rejected.

#### 3.3.1. Day of Measurement

In this case, we will observe the impact of two factors over the course of the day: Time and Site on cumulative values of EF strength. The results of the analysis of variance for individual days of measurement are shown in Table 6.

**Figure 7:**
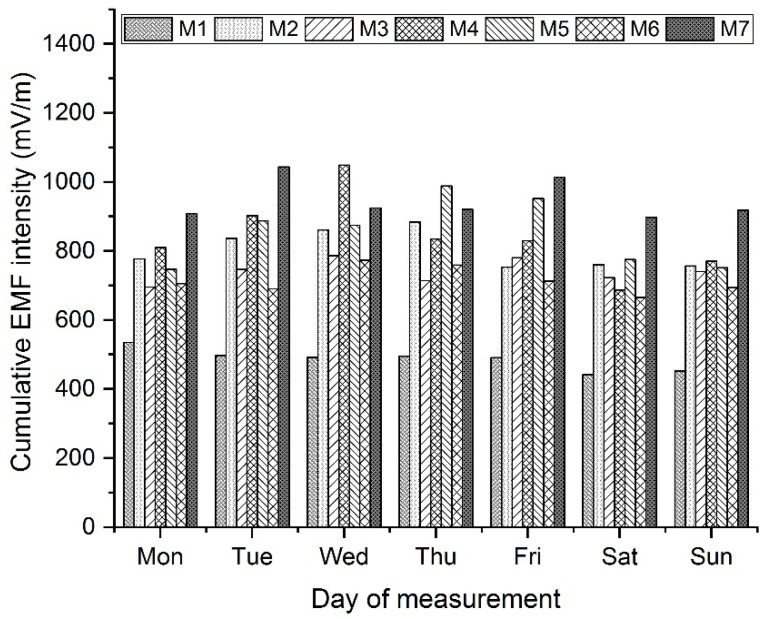
Average values of cumulative value of EF strength measured during the day at a given measurement site.

The results of the analysis of variance show that on each day of measurement, the measurement site has a statistically significant effect on the resulting cumulative values of EF strength. On the five days of the measurement (Tuesday, Wednesday, Thursday, Friday, and Sunday), the impact of the measuring site $\eta^{2}$ is approximately 50%. If the measurement is performed on Wednesday or Thursday, another factor—time of measurement—also affects the values (although the impact is low, about 15%). Results of coefficients $\eta^{2}$ indicate a medium to large effect of the observed factor. A graphical representation of average values of the cumulative EF strength measured during the day at individual measurement sites (factor is the measuring site) is shown in Figure 7.

The results from the ANOVA method indicate a significant impact of the measurement site on the resulting cumulative EF strength value. We used Scheffe’s method to determine between which two levels of the site factor (i.e., between which two measurement sites) there are statistically significant differences during a given day. The results of the testing are shown in Table 7. 

Using the Scheffe’s method, no statistically significant differences between the pairs were confirmed for the time factor.

These results were expected. The significance of the analysis lies in the confirmation of this assumption and especially in its quantification. A difference of 20–25% has been demonstrated; these values should be considered when planning measurements. It is also necessary to take into account the absolute values of the EF strength and its comparison with the action value set by the legislation.

#### 3.3.2. Time (Hour) of Measurement

In this case, we will observe the effect of two factors—site and day on the cumulative EF strength values over the course of two hours. The results of the analysis of variance for individual times of measurement are given in Table 8.

The analysis of variance shows that at each specified time of measurement, the measurement site has a statistically significant effect on the resulting values of the cumulative value of the EF strength. At two times (2 p.m., 8 p.m.) the effect of the factor is even more than 60%. If the measurement is performed at 10 a.m. or 8 p.m., the effect of another factor is shown, namely the day of measurement. The resultant coefficients $\eta^{2}$ indicate medium to large effects of the factor being studied.

**Figure 8:**
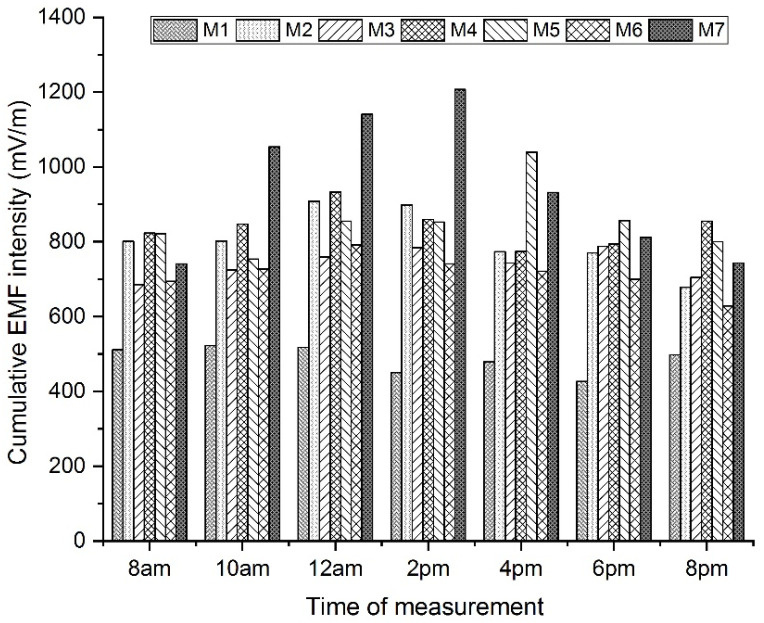
Average values of cumulative EF strength value measured at a given hour at a given measuring site.

A graphical representation of the average values of cumulative EF strength measured at each hour at all measuring sites is shown in Figure 8.

The results of Scheffe’s method, which was used to determine pairs of measurement sites with statistically significant differences, are shown in Table 9.

Through Scheffe’s method, statistically significant differences in the cumulative EF strength value between the pairs for the day of measurement factor at the time of measurement at 8 p.m. were not confirmed.

The results of our research are consistent with the references used. As in [17], our values were below the thresholds set out in the legislation. We also share the opinion of the authors [26] on the importance of continuous monitoring of radiofrequency EMF exposure.

### 3.4. Analyze Interior Measurement Values

Based on statistical analysis, dominant time intervals were predicted during which, based on statistical significance, the highest load of the environment with electromagnetic fields can be expected. Based on the analysis of the measurement results, the measurements were taken at 10 a.m. on Thursday. As part of the experiment, we measured the intensity of the electric field in a building that is in the immediate vicinity of the MBTS. Due to the requirement to minimize the intensity of electric fields penetrating the interior mainly through window fillings and the nature of the experiment, exterior blinds based on an aluminum alloy with a weight of 2.75 g/cm^3^ were designed. The blinds are of a more massive construction with an average material thickness of 0.1 cm, a slat width of 8 cm, and an overlap of 10% of the area when closed. The areal weight of the blind with the considered distance between the slats is 3.02 kg/m^3^. Based on the experiments carried out so far, the distance between the slats does not have a significant effect on the damping effects of the blinds. The specific weight of the metal and the areal weight of the blind have a decisive influence on the damping effects. From a design point of view, it was not possible to conduct an experiment with another type of material (Fe, brass, etc.), because only blinds based on aluminum alloys are commercially available. Other types of materials, especially plastics, were not used in the experiment, as their ability to absorb electromagnetic fields is extremely low.

**Figure 9:**
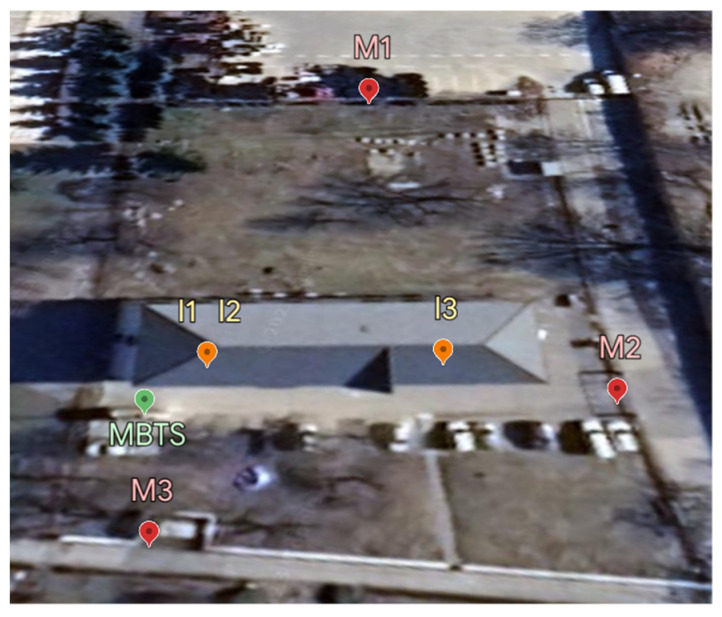
Location of interior measurement sites.

Measurements were made at three measurement points in the front part of the building, which is oriented towards the base station of the mobile operator. Figure 9 shows the location of the measuring points labeled interior I1–I3. The figure also shows the location of MBTS and measuring points in the exterior of M1–M3.

**Figure 10:**
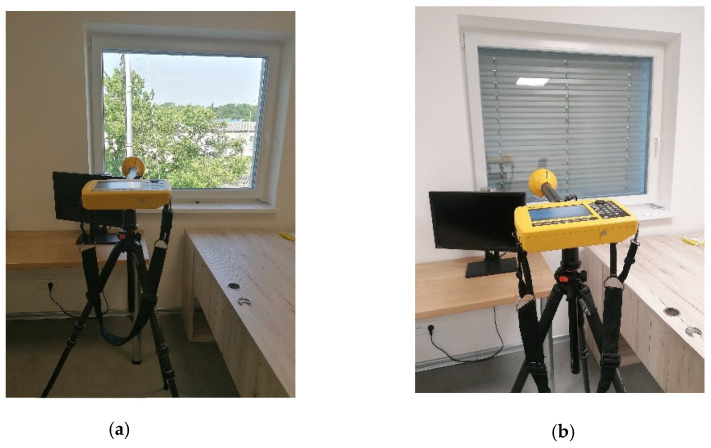
Location of interior measurement site I2 during the measurement. (**a**) Measurement site I2—2nd situation; (**b**) Measurement site I2—3rd situation.

The measuring point I1 was located on the first floor of the investigated building overlooking the base station. The measuring points I2 and I3 were located on the second floor of the multifunctional building towards the EMF source, Figure 9. A direct view of the MBTS construction is possible from the room. We measured the intensity of the electric field in the internal environment of the building in three different situations. The first measurement was performed under conditions where the base station was placed on the roof of the opposite building 110 m away. The second electric field strength measurement was performed after placing the MBTS near the investigated building before installing the shading blinds. The third measurement was taken when applying the blinds and closing them 100%. Figure 10 shows the location of the measurement point I2 during the second and third measurement situations. 

**Figure 11:**
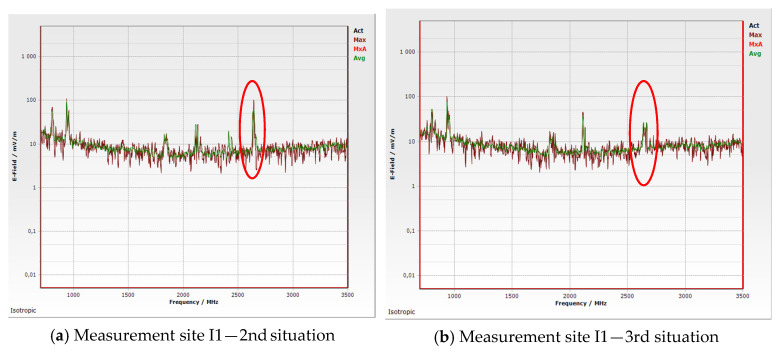
Measurement output—I1 frequency spectrum (Thursday at 10 a.m., instrument generated graph). (**a**) 2nd situation; (**b**) 3rd situation.

Figure 11 shows examples of frequency spectra that we analyzed during the experiment. At the frequency of 2600 MHz, the most pronounced decrease in the measured value of the electric field intensity occurred when measuring with applied blinds. It is necessary to be aware of the logarithmic character of the electric field intensity scale. The decrease in values occurred in the entire considered spectrum, not only at the indicated frequency of 2600 MHZ, where the decrease was dominant.

The output from the measurements are the cumulative values of the electric field intensity for all three measurement variants in the three selected measurement locations. In Table 10 we have an indication of the measured value in units of mV/m.

**Figure 12:**
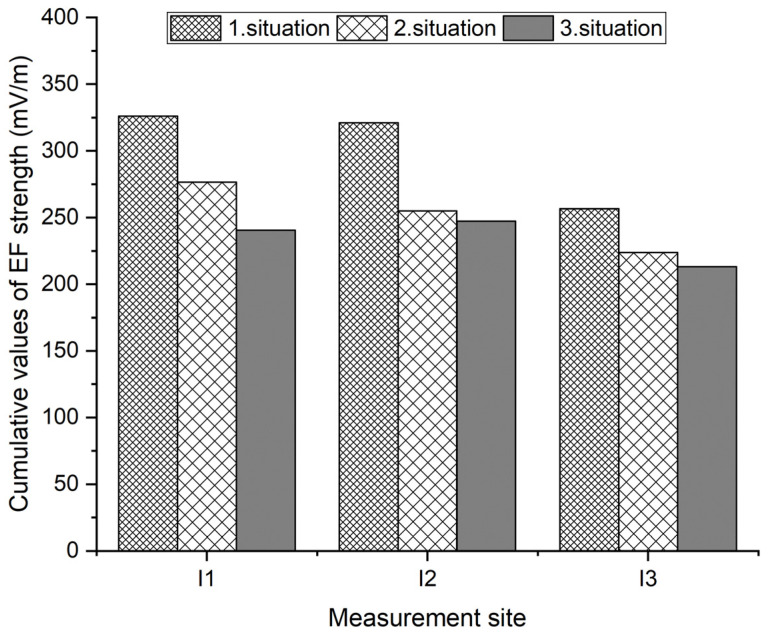
Cumulative values of EF strength measured at a given measuring site.

A graphical representation of the cumulative values of EF strength measured at 10 a.m. on Thursday at all measuring sites is shown in Figure 12.

The direction (azimuth) of the transmitting antennas was the same in situations 1, 2, and 3. The highest EMP values were measured in situation 1, when the base station was placed on the roof of the building opposite the assessed building at 110 m. In situation 2, when the base station on the roof of the building opposite the assessed building at 110 m was removed and the MBTS base station was in operation, the measured values decreased significantly even in view of the fact that the distance of the MBTS decreased to approx. 15 m. The reason for the decrease is the in situ window filling in the transmitting lobe of the antenna. In situation 3, when damping blinds were installed, there was a further reduction in the measured values, which is caused by the damping properties of the blinds, which are decisively influenced by the properties of the used metal materials and the geometric characteristics of the blinds.

## 4. Conclusions

The authors present part of the experimental results of the field measurements carried out in the interior of a residential building situated near the mobile operator station, that aimed to find out the efficiency of the shielding materials placed in the windows and observe the time of occurrence of maximum values of EMFs during the day. 

The research results obtained using statistical methods (ANOVA, Scheffe’s method) show that there are both a time of measurement and a day of measurement, which can show statistically significant changes in the values of the measured EMF intensity.

The conclusions of this experiment are valid for the selected location. For EMF intensity monitoring, a site where many companies are based was chosen, where intensive use of the services of mobile operators (mobile calls and data transmission) is assumed. It is also true that increased data transfer requirements are mainly during working hours and weekdays on this site.

In the next stage, the same methodology will be used to assess a site with a residential function only, in order to confirm or not confirm the hypothesis that the site has an impact on the dependencies considered.

The use of selected glazing materials—an aluminum alloy with a volume weight of 2.75 g/cm^3^ and an area weight of 3.02 kg/m^3^ with the specified geometry caused a significant decrease in the measured values of the electric field in the interior of the building under consideration. For this reason, we can consider them as suitable application variants for situations where it is necessary to reduce the risk of the influence of electric fields on the occupants in the interior. This requirement often occurs in practice even though the measured values are well below the limits of permissible values. These requirements are caused by the effort to improve the quality of the living environment; part of the requirements is caused by the requirements of residents who are highly sensitive to electromagnetic fields.

The application of metal materials represents a simple and effective solution to the problem of protecting residents from the effects of electric fields. Their advantages are accumulated positive properties such as shading from the sun, protection of window fillings, thermal insulation properties, aesthetic properties, and energy saving when the smart building system is introduced.

## Figures and Tables

**Table 1:** Distance of measurement sites from MBTS.

	M1	M2	M3	M4	M5	M6	M7
MBTS	65 m	45 m	25 m	90 m	140 m	124 m	255 m

**Table 2:** Cumulative EF strength values (mV/m) at measurement sites M1 and M7.

Measurement Day	Time of Measurement/Measurement Site													
	8 a.m.		10 a.m.		12 a.m.		2 p.m.		4 p.m.		6 p.m.		8 p.m.	
	M1	M7	M1	M7	M1	M7	M1	M7	M1	M7	M1	M7	M1	M7
Monday	511.1	740.4	526.8	946.5	557.1	1623	558.7	931	458.8	582.4	529.8	762	602.4	777.6
Tuesday	549.8	727.2	461.8	1274	484.6	1149	407	1517	698.4	835.5	388.3	1089	486.4	713.5
Wednesday	480.5	829.1	523.6	1062	709.4	1099	374.2	1343	432.5	718.4	475.7	735.1	448.8	690.3
Thursday	569.6	748.6	595.8	1188	560.9	947.5	464.1	1186	440	960.3	423.9	726.8	411.7	686.6
Friday	466.5	697.6	561.5	1240	514.6	1083	590	1360	425.3	1271	393	751.3	485.3	697.2
Saturday	550.9	762.5	478.6	744.9	392.3	1096	348	1035	433.4	1003	369.1	875.2	520.9	767.2
Sunday	449	679.1	506.3	918.6	405.8	988	410.4	1081	462.8	1150	407.9	742.6	527.9	870.7

**Table 3:** Descriptive statistics of cumulative EF strength values (mV/m)—day of measurement.

Characteristics	Day of Measurement						
	Mon	Tue	Wed	Thu	Fri	Sat	Sun
Mean	740.3	800.7	823.2	799.6	790.7	705.6	726.7
Standard deviation	194.4	230.9	229.3	214.4	228.9	199.9	177.6
Maximum	1623.0	1517.0	1343.0	1267.0	1360.0	1371.0	1150.0
Minimum	458.8	388.3	374.2	411.7	393.0	348.0	405.8
Range (Max-Min)	1164.2	1128.7	968.8	855.3	967.0	1023.0	744.2

**Table 4:** Descriptive statistics of cumulative EF strength values (mV/m)—time of measurement.

Characteristics	Time of Measurement						
	8 a.m.	10 a.m.	12 a.m.	2 p.m.	4 p.m.	6 p.m.	8 p.m.
Mean	725.5	775.6	843.7	827.8	780.1	735.2	700.9
Standard deviation	166.4	212.7	242.9	257.1	231.4	194.3	137.7
Maximum	1230.0	1314.0	1623.0	1517.0	1371.0	1267.0	977.0
Minimum	449.0	461.8	392.3	348.0	425.3	369.1	411.7
Range (Max–Min)	781.0	852.2	1230.7	1169.0	945.7	897.9	565.3

**Table 5:** Overview of variables.

Variables	Description
Factors	
measurement site	Seven measurement sites (M1, M2, M3, M4, M5, M6, M7)
day of measurement	7 days of the week (D1 = Monday, D2 = Tuesday, D3 = Wednesday, D4 = Thursday, D5 = Friday, D6 = Saturday, D7 = Sunday)
time of measurement	Seven measurements (H1 = 8 a.m., H2 = 10 a.m., H3 = 12 a.m., H4 = 2 p.m., H5 = 4 p.m., H6 = 6 p.m., H7 = 8 p.m.)
Measured (output) variable	
intensity	Cumulative value of electromagnetic field intensity (mV/m)

**Table 6:** Testing results—day of measurement (factors: measurement site, time of measurement) α = 0.05.

	Monday	Tuesday	Wednesday	Thursday	Friday	Saturday	Sunday
Factor/*p*-value							
Site: *p*-value	0.012	9.10^−6^	9.10^−6^	7.10^−6^	3.10^−5^	3.10^−4^	2.10^−6^
Time: *p*-value	0.328	0.063	0.036	0.047	0.147	0.187	0.065
Conclusion							
Site:	Rejected	Rejected	Rejected	Rejected	Rejected	Rejected	Rejected
Time:	Not rejected	Not rejected	Rejected	Rejected	Not rejected	Not rejected	Not rejected
Effect of the factor $\eta^{2}$							
Site:	30.9%	50.8%	49.6%	50.8%	48.7%	42.6%	53.9%
Time:	11.5%	13.3%	15.0%	14.1%	11.7%	11.9%	12.4%

**Table 7:** Multiple Comparisons result—day of measurement (α = 0.05).

Day	Factor	Pairs—Significant Differences
Monday	Site	M1–M7
Tuesday	Site	M1–M2, M1–M4, M1–M5, M1–M7, M6–M7
Wednesday	Site	M1–M2, M1–M4, M1–M5, M1–M7
Thursday	Site	M1–M2, M1–M4, M1–M5, M1–M7
Friday	Site	M1–M4, M1–M5, M1– M7
Saturday	Site	M1–M2, M1– M7
Sunday	Site	M1–M2, M1–M3, M1–M4, M1–M5, M1–M7

**Table 8:** Testing results—time of measurement (factors: measurement site, day of measurement) α = 0.05.

	8 a.m.	10 a.m.	12 a.m.	2 p.m.	4 p.m.	6 p.m.	8 p.m.
Factor/*p*-value							
Site: *p*-value	8 × 10^−4^	1 × 10^−6^	3 × 10^−6^	5 × 10^−9^	4 × 10^−5^	1 × 10^−4^	2 × 10^−8^
Day: *p*-value	0.099	2 × 10^−3^	0.073	0.07	0.400	0.573	0.048
Conclusion							
Site:	Rejected	Rejected	Rejected	Rejected	Rejected	Rejected	Rejected
Day:	Not rejected	Rejected	Not rejected	Not rejected	Not rejected	Not rejected	Rejected
Effect of the factor $\eta^{2}$							
Site:	38.7%	49.2%	53.5%	66.8%	50.7%	48.0%	63.4%
Day:	15.1%	21.6%	12.2%	8.7%	7.5%	6.1%	10.4%

**Table 9:** Multiple Comparisons result—time of measurement (α = 0.05).

Time	Factor	Pairs—Significant Differences
8 a.m.	Site	M1–M2, M1–M4, M1–M5, M1–M7
10 a.m.	Site	M1–M2, M1–M4, M1–M7, M3–M7, M5–M7, M6–M7
	Day	Saturday–Thursday, Saturday–Friday
12 a.m.	Site	M1–M2, M1–M4, M1–M5, M1–M7, M3–M7, M6–M7
2 p.m.	Site	M1–M2, M1–M3, M1–M4, M1–M5, M1–M7, M3–M7, M5–M7, M6–M7
4 p.m.	Site	M1–M5, M1–M7
6 p.m.	Site	M1–M2, M1–M3, M1–M4, M1–M5, M1–M7
8 p.m.	Site	M1–M2, M1–M3, M1–M4, M1–M5, M1–M7

**Table 10:** Cumulative EF strength values (mV/m) at interior measurement sites.

Measurement Site	I1	I2	I3
1st measurement situation	326.1	321.0	256.6
2nd measurement situation	276.6	255.0	223.7
3rd measurement situation	240.9	247.3	213.1

## Data Availability

The data presented in this study are available on request from the corresponding author.
